# Targeting PML-RAR**α** and Oncogenic Signaling Pathways by Chinese Herbal Mixture *Tien-Hsien* Liquid in Acute Promyelocytic Leukemia NB4 Cells

**DOI:** 10.1093/ecam/nep165

**Published:** 2011-02-20

**Authors:** Chih-Jung Yao, Chia-Ming Yang, Shuang-En Chuang, Jiann-Long Yan, Chun-Yen Liu, Suz-Wen Chen, Kun-Huang Yan, Tung-Yuan Lai, Gi-Ming Lai

**Affiliations:** ^1^Cancer Center, Taipei Medical University-Wan Fang Hospital, Taiwan; ^2^Formosa Cancer Foundation, Taipei, Taiwan; ^3^National Institute of Cancer Research, National Health Research Institutes, Zhunan, Taiwan; ^4^Department of Life Science, National Tsing Hua University, Hsinchu, Taiwan; ^5^School of Post-Baccalaureate Chinese Medicine, China Medical University, Taichung, Taiwan; ^6^Department of Chinese Internal Medicine, China Medical University Hospital, Taichung, Taiwan

## Abstract

*Tien-Hsien* Liquid (THL) is a Chinese herbal mixture that has been used worldwide as complementary treatment for cancer patients in the past decade. Recently, THL has been shown to induce apoptosis in various types of solid tumor cells *in vitro*. However, the underlying molecular mechanisms have not yet been well elucidated. In this study, we explored the effects of THL on acute promyelocytic leukemia (APL) NB4 cells, which could be effectively treated by some traditional Chinese remedies containing arsenic trioxide. The results showed THL could induce G2/M arrest and apoptosis in NB4 cells. Accordingly, the decrease of cyclin A and B1 were observed in THL-treated cells. The THL-induced apoptosis was accompanied with caspase-3 activation and decrease of PML-RAR**α** fusion protein. Moreover, DNA methyltransferase 1 and oncogenic signaling pathways such as Akt/mTOR, Stat3 and ERK were also down-regulated by THL. By using ethyl acetate extraction and silica gel chromatography, an active fraction of THL named as EAS5 was isolated. At about 0.5–1% of the dose of THL, EAS5 appeared to have most of THL-induced multiple molecular targeting effects in NB4 cells. Based on the findings of these multi-targeting effects, THL might be regarding as a complementary and alternative therapeutic agent for refractory APL.

## 1. Introduction

Due to the severe side effects and limits of therapeutic efficacy of conventional cancer treatment, the use of complementary and alternative medicine (CAM) for improving clinical outcome and reducing side effects is prevalent worldwide. Among the various CAM treatment modalities, traditional Chinese medicine (TCM) with anticancer activity is the most popular choice. The *Tien-Hsien* Liquid (THL) is a Chinese herbal mixture, which has been used as a complementary anticancer agent for more than 10 years. It mainly consists of extracts from 14 Chinese medicinal herbs: *Cordyceps sinensis* (CS), *Oldenlandia diffusa* (OD), *Indigo pulverata levis* (IPL; also known as *Indigo naturalis*), *Polyporus umbellatus* (PU), *Radix astragali* (RA), *Panax ginseng* (PG), *Solanum nigrum L.* (SNL), *Pogostemon cablin* (PC), *Atractylodis macrocephalae rhizoma* (AMR), *Trichosanthes radix* (TR), *Clematis radix* (CR), *Margarite* (M), *Ligustrum lucidum Ait* (LLA) and *Glycyrrhiza radix* (GR) [1]. The biological activities of these herbs have been reported individually, including antioxidation, antimutagenesis, immunomodulation as well as cytostatic or cytotoxic effects and so forth. Recently, THL has been shown to induce apoptosis in many types of cancer cells and activate caspase-8, -9 and -3 in H1299 lung cancer cells [2].

Although those studies provided some information about the molecular basis of THL-induced anticancer activities, it is worthwhile to investigate additional molecular mechanisms. One clinically successful example of TCM therapy is the effective treatment of acute promyelocytic leukemia (APL) by some traditional Chinese remedies containing arsenic compounds [3, 4]. We attempted to explore the effects of THL on the NB4 APL cells and study the related molecular mechanisms.

Cell-cycle regulation has been suggested as a target for cancer therapy [5, 6]. Mitotic arrest of cell cycle was an important mechanism for arsenic trioxide (ATO)-induced apoptosis in APL cells [7]. There may also be a tight link between THL-induced apoptosis and arrest of cell cycle. Therefore, we investigated the influence of THL on cell-cycle regulation and the related cyclins and cyclin-dependent kinase (CDK) in NB4 APL cells.

APL, a particular subtype of acute myeloid leukemia (AML) with a distinct cytologic morphology (M_3_ and M_3_ variant in French-American-British (FAB) classification), is characterized by a chromosome translocation t (15; 17), which results in the rearrangement of promyelocytic leukemia (*PML*) gene and retinoic acid receptor-*α* (*RAR*α**) gene, and then the expression of PML-RAR*α* fusion protein [3]. It is commonly accepted that PML-RAR*α* fusion protein forms the stable complexes with DNA methyltransferases (DNMTs) and with corepressors, such as SMRT/NCoR/histone deacetylase, to repress the RARE (retinoic acid response element)-containing genes [8]. On the other hand, it is reported that up-regulated DNMTs may contribute to the pathogenesis of leukemia by inducing aberrant regional hypermethylation [9]. Effective agents for APL such as all-trans retinoic acid (ATRA) and ATO are both able to degrade the PML-RAR*α* [10, 11] and inhibit DNMT [12, 13]. To more clarify the effects of THL on APL cells, its influences on PML-RAR*α* and DNMT1, the most important DNMT for maintaining the aberrant methylation in cancer cells [14], were investigated in this study.

Additionally, in leukemia and other hematopoietic disorders, the Akt, Stat and extracellular signal-regulated kinase (ERK) signaling pathways are frequently activated by upstream mutations of cytokine receptors, aberrant chromosomal translocations as well as other genetic problems [15], resulting in suppression of apoptosis and deregulation of proliferation [16]. Targeting these oncogenic signaling pathways for effective leukemia therapy thus had been proposed [15]. To understand the effects of THL on APL cells more, its effects on these signaling molecules were also studied.

In order to improve the potency of THL, we try to find the active components by solvent extraction and silica gel chromatography. An active fraction named as EAS5 was isolated, which appears to have much higher potency in apoptosis induction than THL. The mechanisms underlying the effects of EAS5 were investigated as well in this study.

## 2. Methods

### 2.1. Cell Culture

Human APL NB4 cells were cultured in RPMI 1640 medium (Gibco, Grand Island, NY, USA) supplemented with 10% fetal bovine serum (Gibco) and 1% Penicillin-Streptomycin-Glutamine (100×) liquid (Gibco). The cells were maintained at 37°C in a humidified atmosphere of 95% air and 5% CO_2_.

### 2.2. THL and Preparation of Active Fraction EAS5

THL was provided by Feida Union Pharmaceutical Manufactory, El Monte, CA. It is an aqueous preparation of herbal mixture and consists mainly of extracts from 14 Chinese medicinal herbs as mentioned above. The original THL aqueous solution was lyophilized, weighed and then stored in −20°C. It was reconstituted with sterile distilled water to prepare the working solutions for experimental use. To isolate the active fraction EAS5, THL was extracted by ethyl acetate. The extract was subjected to silica gel chromatography eluted by the gradient ratios of ethyl acetate/*n*-hexane and then gradient ratios of methanol/ethyl acetate. The fifth fraction EAS5 eluted by ethyl acetate/*n*-hexane (80/20), was found to be the most potent fraction by its growth-inhibition activity in various cancer cell lines, including NB4 cells. Similarly, EAS5 was lyophilized and then weighed to prepare a 50 mg mL^−1^ stock solution in dimethyl sulfoxide (DMSO) and kept in −20°C. Before being added to cells, the EAS5 stock solution was diluted with PBS to prepare the working solutions. The final DMSO concentrations were all less than 0.1% when EAS5 was added to cells.

### 2.3. Reagent and Antibodies

The ATRA and antibody against *β*-actin (A5441) were purchased from Sigma Chemical Co. (St. Louis, MO, USA). Primary antibodies against cyclin A (SC-239), cyclin B1 (SC-245), cyclin D1 (SC-8396), Cdc2 (sc-54), CDK2 (SC-163), PML (SC-5621), DNMT1 (SC-20701), Stat3 (SC-8019), phosphorylated Stat3 (SC-7993), ERK (sc-94), phosphorylated ERK (SC-7383) and glyceraldehyde-3-phosphate dehydrogenase (GAPDH) (SC-47724) were purchased from Santa Cruz Biotechnology (Santa Cruz, CA, USA). Primary antibodies against caspase-3 (#9661), Akt (#9272), phosphorylated Akt (#9271), mTOR (#2972) and phosphorylated mTOR (#2971) were purchased from Cell Signaling Technology (Beverly, MA, USA). Primary antibody against p21 (05-345) was purchased from Upstate Biotechnology (Lake Placid, NY, USA). Primary antibody against RAR*α* (PC92L) was purchased from Calbiochem (San Diego, CA, USA).

### 2.4. Cell Viability Assay

NB4 cells were seeded in 10 cm dishes at a density of 10^6^ cells/10 cm dish for 24 h and then treated with drugs or PBS for 3 days. On the day of harvest, the cell viability was determined by trypan blue exclusion.

### 2.5. Analysis of Sub-G_1_ Apoptotic Population and Cell-Cycle Distribution

One day after being seeded in a six-well plate (10^5^ cells mL^−1^, 2 mL well^−1^), the NB4 cells were incubated with THL or EAS5 for the time intervals indicated in the figure legends. The control groups were treated with phosphate-buffered saline (PBS) only. At harvest, cells were fixed in ice-cold 70% ethanol and stored at −20°C. Cells were then washed twice with ice-cold PBS and then incubated with RNase and DNA intercalating dye propidium iodide (50 *μ*g mL^−1^). The percentages of sub-G_1_ apoptotic population and cell-cycle distribution were then analyzed with a flow cytometer (BD Biosciences, San Jose, CA, USA).

### 2.6. Western Blot

Western blot was used to examine the THL-induced changes of caspase-3, cyclins, CDKs, PML-RAR*α* and DNMT1 proteins, and antibodies against specific phosphorylated proteins were employed to analyze the activities of affected signaling pathways. Cells were seeded in 10cm dishes at a density of 10^6^ cells/10 cm dish for 24 h and then treated with drugs or PBS for 3 days. On the day of harvest, the whole-cell lysates were prepared with radioimmunoprecipitation buffer containing 50 mM of Tris-HCl (pH 7.4), 150 mM of NaCl, 1% Nonidet-P40, 0.25% sodium deoxycholate, 1 mM of ethylenediaminetetraacetic acid (EDTA), 1 mM of Na_3_VO_4_, 1 mM of NaF, 1 mM of phenylmethanesulfonyl fluoride and 1 : 100 diluted protease inhibitors mixture (Sigma). The protein extracts were subjected to sodium dodecyl sulfate-polyacrylamide gel electrophoresis (SDS-PAGE) and transferred to polyvinylidene difluoride membrane (Bio-Rad, Richmond, CA, USA) by electroblotting. The membranes were blocked with 5% BSA in TBST (25 mM Tris-HCl, 125 mM NaCl, 0.1% Tween 20) for 1 h at room temperature and probed with primary antibody overnight at 4°C and then with horseradish peroxidase–conjugated secondary antibody for 1 h at room temperature. The immune complexes were visualized using the luminol reagent (Santa Cruz Biotechnology, Santa Cruz, CA, USA) according to the manufacturer's instructions.

## 3. Results

### 3.1. THL Induced Apoptosis of NB4 Cells

**Figure 1:**
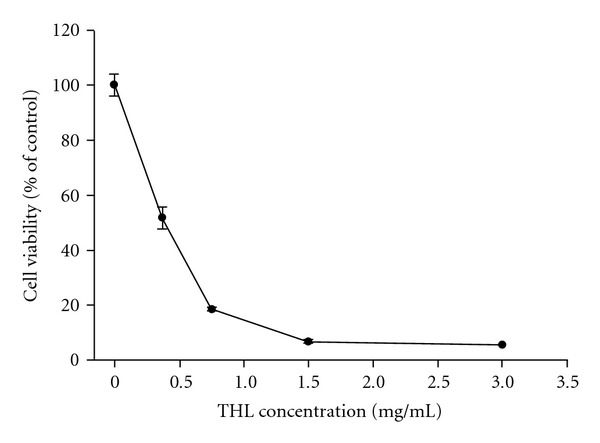
Effects of THL on the proliferation of NB4 leukemia cells. The cells were treated with increasing concentrations of THL (0.375–3 mg/mL) or PBS only for 72 h, and cell viability was determined by trypan blue exclusion. Data (mean ± SE, duplicate) are expressed as a percentage of the PBS-treated control. SE: standard error.

THL dose-dependently decreased the viability of NB4 cells (Figure 1). When treated with 0.375 mg mL^−1^ of THL for 72 h, only 51.49% of NB4 leukemia cells survived, as compared with control. At the dose of 1.5 mg mL^−1^, only 6.71% of cells survived, the IC50 was *∼*0.353 mg mL^−1^.

**Figure 2:**
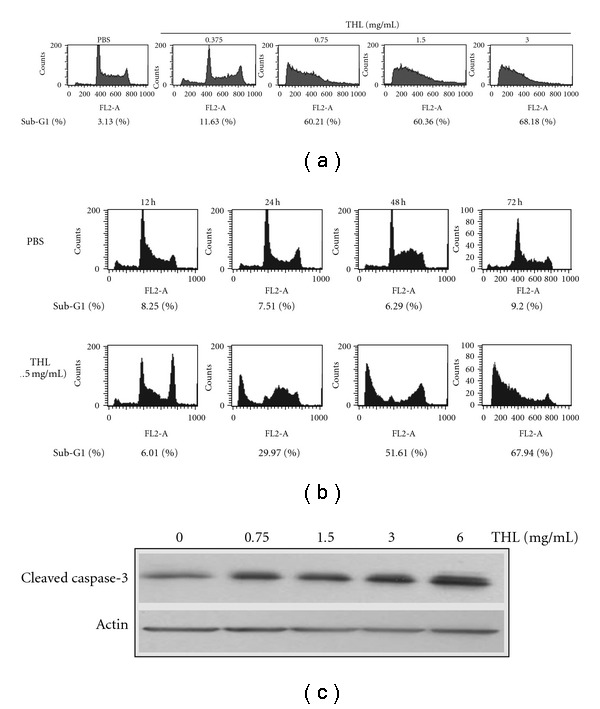
Effects of THL on the apoptosis induction of NB4 cells. (a) THL induced apoptosis of NB4 cells in a dose-dependent manner after 72 h of treatment. (b) The time course of THL (1.5 mg/mL)-induced apoptosis of NB4 cells. The apoptotic sub-G_1_ populations in the flow cytometric analysis histograms were obtained as measures of apoptosis and the percentages of them were shown below the histograms. (c) The protein level of cleaved/activated caspase-3 of NB4 cells was increased by THL after 72 h of treatment. The cells were treated with PBS or THL (0.375–3 mg/mL) for 72 h and then harvested for flow cytometry (a and b) and western blot (c) analysis.

By flow cytometry analysis, a substantial amount of apoptotic cells with subG1 DNA content could be observed in THL-treated cells. As shown in Figure 2(a), THL markedly increased the percentage of apoptotic population in a dose-dependent manner. After 3 days of treatment, a total of 11.63% of subG1 population could be induced by THL at dose of 0.375 mg mL^−1^, compared with the 3.13% of PBS control group. When the dose of THL was greater than 0.75 mg mL^−1^, more than 60% of subG1 population was observed.

The time course of THL (1.5 mg mL^−1^)-induced apoptosis was shown in Figure 2(b). A total of 29.97% and 67.94% of subG1 population were found in THL (1.5 mg mL^−1^)-treated NB4 cells after 24 and 72 h treatment, respectively. In accordance with that of previous report [2], the THL-induced apoptosis in NB4 cells was accompanied with the activation of caspase-3. The cleaved (activated) caspase-3 was dose-dependently increased by THL after 72 h treatment (Figure 2(c)).

### 3.2. THL Induced G2/M-Phase Arrest

**Figure 3:**
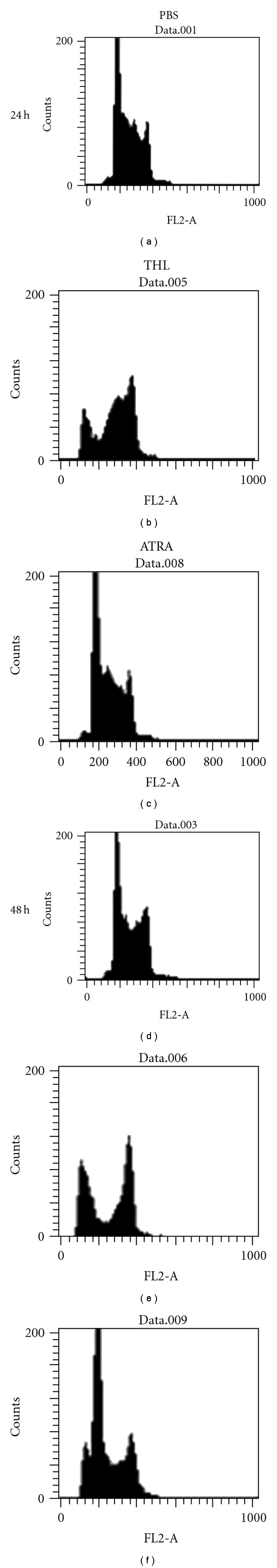
Effects of THL on the cell-cycle distribution of NB4 cells. The cell-cycle distributions were analyzed in the flow cytometric analysis histograms of THL (1.5 mg/mL)- or ATRA (0.25 *μ*M)-treated NB4 cells after 24 and 48 h of treatment. THL induced G2/M arrest in NB4 cells, in contrast to the ATRA-induced G1 arrest. The percentages of cells in different phases of the cell cycle are listed in Table 1.

In addition to subG1 apoptotic population, the increase of G2/M-phase population was observed in THL-treated cells. This phenomenon was different from that induced by ATRA, a common agent for treating APL. As shown in Figure 3 and Table 1, the pattern of cell-cycle distribution induced by THL was quite different from that induced by ATRA. At dose of 1.5 mg mL^−1^, THL increased the percentage of cells in the G2/M phase in a time-dependent manner. After 24 and 48 h of THL treatment, the percentage of G2/M-phase population was markedly increased from 19.21 and 23.27% of PBS control group up to 50.28 and 55.93%, respectively. In contrast, ATRA primarily increased the G1-phase population (Table 1). After 24 and 48 h of ATRA treatment, the percentage of G1-phase population was increased from 48.36 and 48.01% of PBS control group up to 53.9 and 60.85%, respectively.

**Figure 4:**
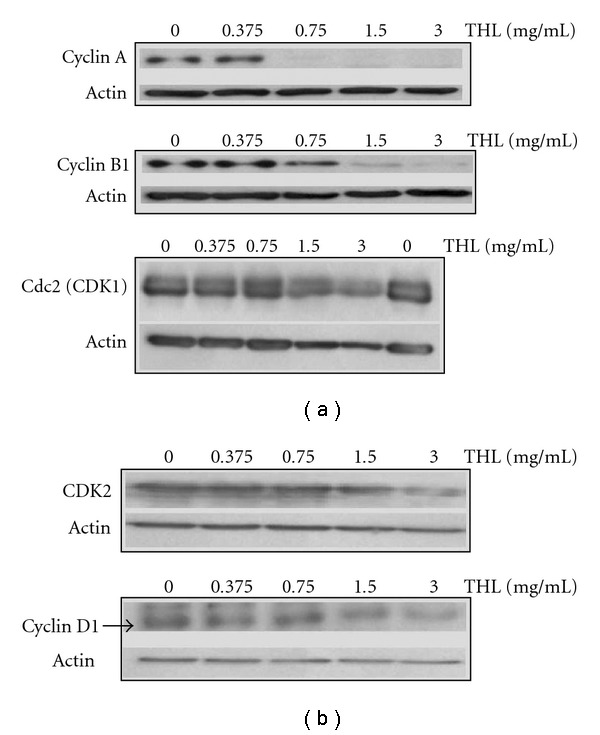
The effects of THL on the cell-cycle regulating proteins of NB4 cells. (a) THL-induced down-regulation of cyclin A and cyclin B1 proteins of NB4 cells in a dose-dependent manner but did not significantly change the Cdc2 (CDK1) level. (b) In contrast to its effects on cyclin A and B1, THL only slightly decreased the protein levels of CDK2 and cyclin D1. The cells were treated with PBS or THL (0.375–3 mg/mL) for 72 h and then harvested. Cell lysates were then prepared for western blot analysis.

**Figure 5:**
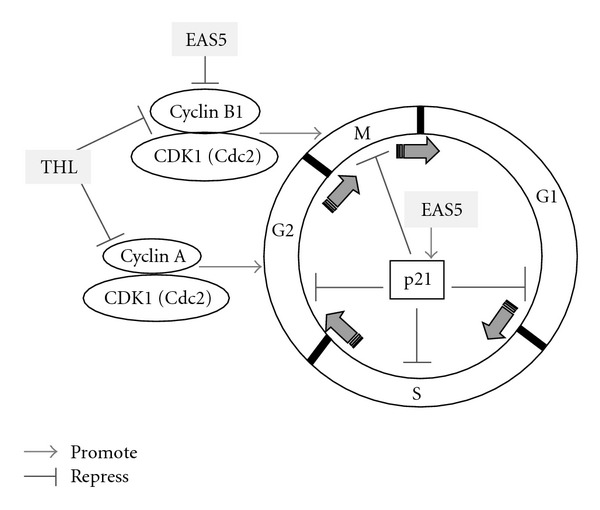
Proposed mechanisms of THL- and its active fraction EAS5-induced G2/M arrest in NB4 cells. The arrow indicates promotion, and the T-shaped bar indicates repression. The data of THL are presented in Figures 3, 4 and Table 1, and the data of EAS5 are presented in Figure 11 and Table 2.

The progression of the cell cycle is mainly controlled by cyclins and CDKs. The cyclin A, cyclin B1 and Cdc2 (CDK1) are required to pass through the G2/M phase [17, 18]. Therefore, we examined whether these proteins were affected by THL. After 72 h of THL treatment, the western blot analysis showed the protein levels of cyclin A and cyclin B1 were markedly and dose-dependently decreased. As shown in Figure 4(a), the protein levels of cyclin A and cyclin B1 were almost completely diminished by THL at doses of 0.75 mg mL^−1^ and 3 mg mL^−1^, respectively. In contrast, the changes of Cdc2 (Figure 4(a)) and other cell-cycle regulating proteins such as cylin D1 and CDK2 were very little. As shown in Figure 4(b), THL only slightly reduced the protein levels of cyclin D1 and CDK2 even up to the dose of 3 mg mL^−1^. The proposed mechanism of THL induced G2/M arrest in NB4 cells was illustrated in Figure 5.

### 3.3. THL Down-Regulated PML-RAR*α* Fusion Protein and DNMT1

**Figure 6:**
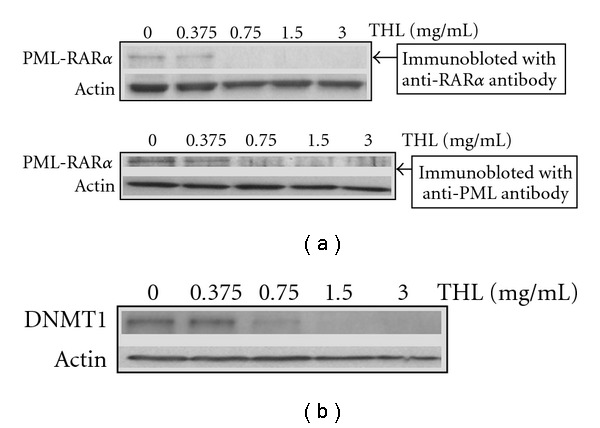
The effects of THL on the PML-RAR*α* fusion protein and DNMT1 of NB4 cells. (a) THL dose-dependently decreased the protein level of PML-RAR*α* detected by antibodies against either RAR*α* or PML. (b) The protein level of DNMT1 was also decreased by THL in the same manner. The cells were treated with PBS or THL (0.375–3 mg/ml) for 72 h and then harvested. Cell lysates were then prepared for western blot analysis.

**Figure 7:**
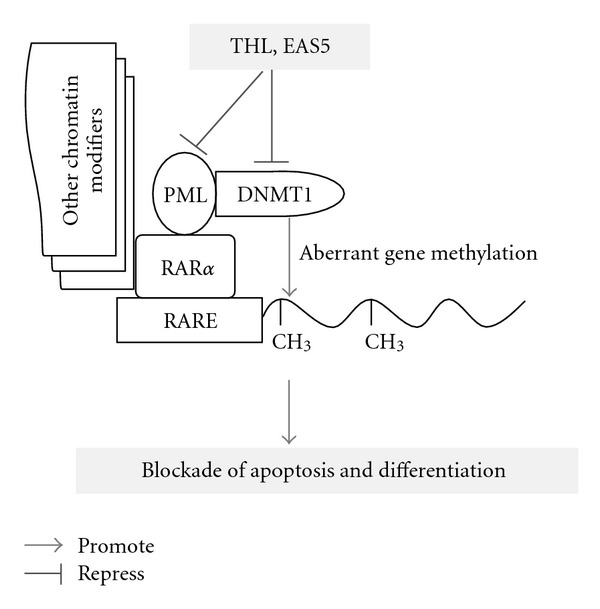
In NB4 cells, PML-RAR*α* expression leads to aberrant gene methylation by recruitment of DNMT1 and other chromatin modifiers. Both the PML-RAR*α* and DNMT1 could be repressed either by THL or its active fraction EAS5, which resulted in apoptosis of leukemia cells. The arrow indicates promotion, and the T-shaped bar indicates repression. The data of THL are presented in Figure 6, and the data of EAS5 are presented in Figure 12.

After 72 h of treatment, THL dose-dependently decreased the PML-RAR*α* fusion protein, which was detected by antibodies against either RAR*α* or PML (Figure 6(a)). THL almost completely diminished the PML-RAR*α* fusion protein at the dose above 0.75 mg mL^−1^. This result coincided with the remarkable apoptosis induction shown in Figure 2(a). As the PML-RAR*α* would cooperated with DNMT to exert its oncogenic effects [8, 13], we examined the DNMT1 of THL-treated NB4 cells as well. After 72 h treatment, the DNMT1 was also markedly down-regulated by THL at the dose of 0.75 mg mL^−1^ (Figure 6(b)). THL substantially decreased both the PML-RAR*α* and DNMT1 proteins of NB4 APL cells. The scheme explaining these effects of THL was illustrated in Figure 7.

### 3.4. THL Inhibited Oncogenic Signaling Pathways

**Figure 8:**
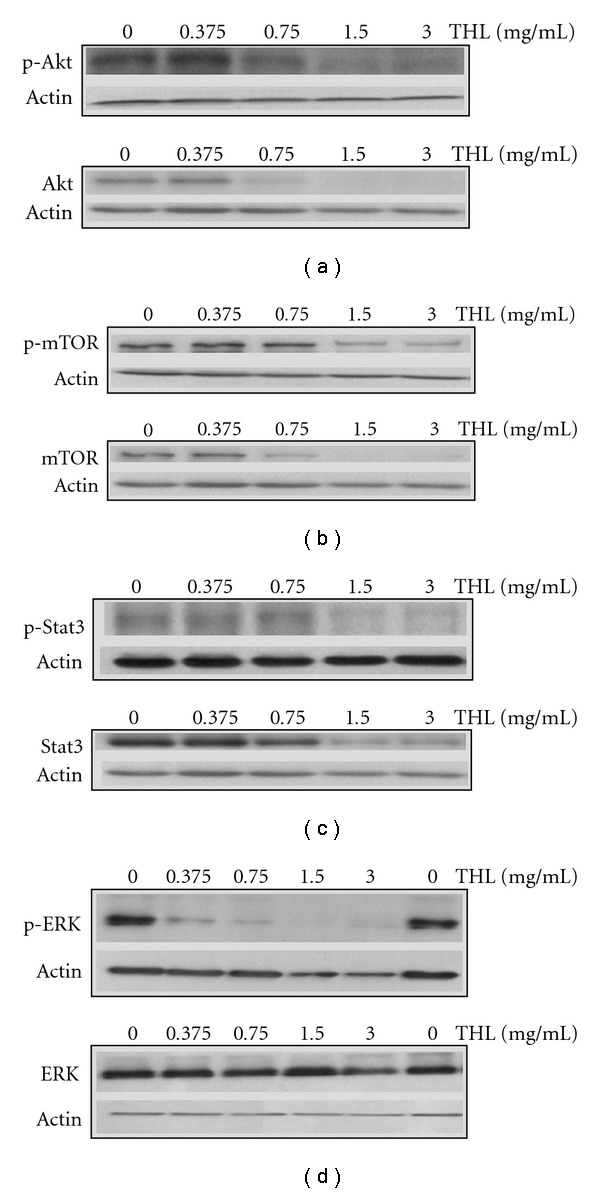
THL-induced inhibition of oncogenic signaling pathways in NB4 cells. Western blot analysis for phosphorylated (p-) and total Akt (a), mTOR (b), Stat3 (c) and ERK (d) proteins of THL-treated NB4 cells. Phosphorylated Akt, mTOR, Stat3 and ERK were all significantly inhibited by treatment with THL. Total protein levels of Akt, mTOR and Stat3 were also significantly decreased by THL, while the total ERK protein was only slightly decreased by the highest dose of THL. The cells were treated with PBS or THL (0.375–3 mg/ml) for 72 h and then harvested. Cell lysates were then prepared for western blot analysis.

It is reported that the proliferation and apoptotic resistance of leukemia cells was closely related to several oncogenic signaling pathways such as Akt/mTOR, Stat3 and ERK [19–24]. In addition to the inhibitory effects on the aberrantly expressed or elevated proteins, we also investigate the influences of THL on these signaling pathways of NB4 cells. After 72 h treatment of NB4 cells with THL, the constitutively phosphorylated Akt protein was decreased in a dose-dependent manner. Indeed, THL decreased not only AKT activity but also down-regulated total AKT protein (Figure 8(a)). Similar results were also observed in the mTOR (mammalian target of rapamycin), an important downstream mediator of Akt, and Stat3. THL dose-dependently decreased both the phosphorylated and total proteins of mTOR and Stat3 in NB4 cells (Figures 8(b) and 8(c)).

**Figure 9:**
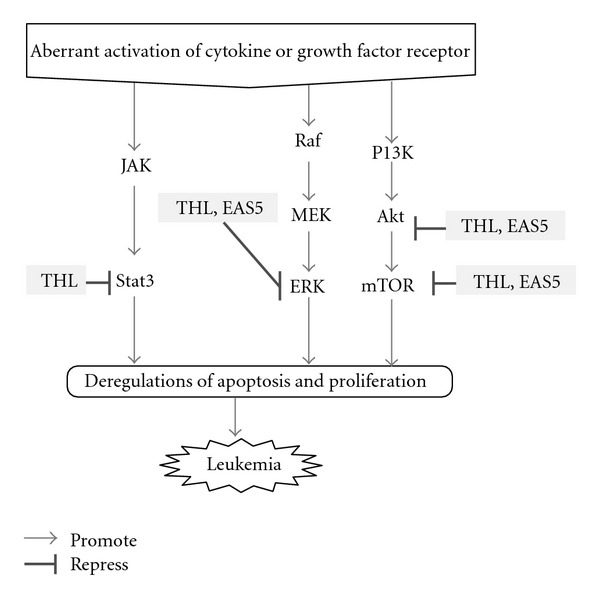
Schematic illustration of the inhibitory effects of THL and its active fraction EAS5 on the oncogenic signaling pathways in NB4 cells. The arrow indicates promotion, and the T-shaped bar indicates repression. The dramatic inhibition on ERK pathway is indicated by a thicker T-shaped bar. The data of THL are presented in Figure 8, and the data of EAS5 are presented in Figure 13.

As shown in Figure 8(d), after 72 h of THL treatment, dramatic inhibition of ERK phosphorylation could be seen at dose above 0.375 mg mL^−1^. However, unlike its effects on other signaling proteins such as Akt, mTOR and Stat3, the total ERK protein level in NB4 cells was only slightly decreased even at the highest dose of THL.  Figure 9 schematically illustrated the effects of THL on the oncogenic signaling pathways in NB4 cells.

### 3.5. Active Fraction EAS5 Exerted Most Molecular Targeting Effects of THL

**Figure 10:**
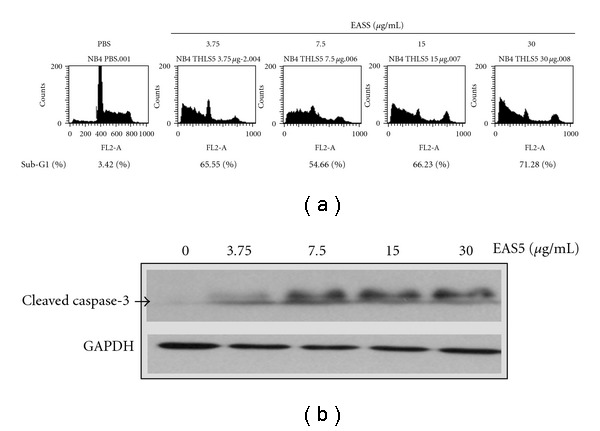
EAS5 induced apoptosis and activated caspase-3 in NB4 cells. (a) EAS5-induced apoptosis of NB4 cells in a dose-dependent manner after 72 h of treatment. The apoptotic sub-G_1_ populations in the flow cytometric analysis histograms were obtained as measures of apoptosis and the percentages of them were shown below the histograms. (b) The protein level of cleaved/activated caspase-3 of NB4 cells was increased by EAS5 after 72 h of treatment. The cells were treated with PBS or EAS5 (3.75–30 *μ*g/mL) for 72 h and then harvested for flow cytometry (a) and western blot (b) analysis. EAS5: active fraction of THL.

**Figure 11:**
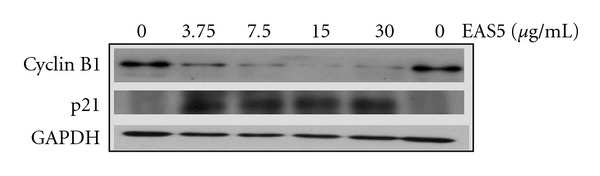
Effects of EAS5 on the protein levels of cyclin B1 and p21 in NB4 cells. EAS5 down-regulated cyclin B1 and elevated p21 protein levels in NB4 cells. The cells were treated with PBS or EAS5 (3.75–30 *μ*g/ml) for 72 h and then harvested. Cell lysates were then prepared for western blot analysis. EAS5: active fraction of THL.

By ethyl acetate extraction and subsequent silica gel chromatography, an active fraction named EAS5 was obtained from THL. At about 0.5–1% of the dose of THL, the active fraction EAS5 could induce apoptosis and G2/M arrest in NB4 cells. After 72 h of treatment, massive apoptosis (Figure 10(a)) and the increase of cleaved caspase-3 (Figure 10(b)) were observed in cells treated with EAS5, even at the lowest dose (3.75 *μ*g mL^−1^). The cell-cycle distributions shown in Figure 10(a) were outlined in Table 2. After 72 h treatment, the percentage of G2/M-phase cells was increased from 14.44% of the PBS control group to 23.07 and 44.14% by EAS5 at doses of 3.75 and 30 *μ*g mL^−1^, respectively. In accordance with the above results, treatment with EAS5 also resulted in a conspicuous decrease of cyclin B1. In addition, a broad-spectrum CDK inhibitor p21 [25] was also markedly increased by EAS5 (Figure 11). The increased p21 would also inhibit the G2/M phase transition by Cdc2 inhibition, which was also illustrated in Figure 5.

**Figure 12:**
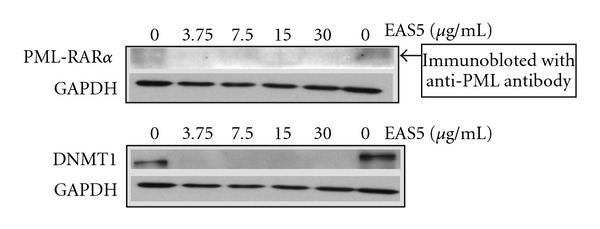
The effects of EAS5 on the PML-RAR*α* fusion protein and DNMT1 of NB4 cells. EAS5 dramatically decreased the protein levels of PML-RAR*α* and DNMT1. The cells were treated with PBS or EAS5 (3.75–30 *μ*g/mL) for 72 h and then harvested. Cell lysates were then prepared for western blot analysis. EAS5: active fraction of THL.

**Figure 13:**
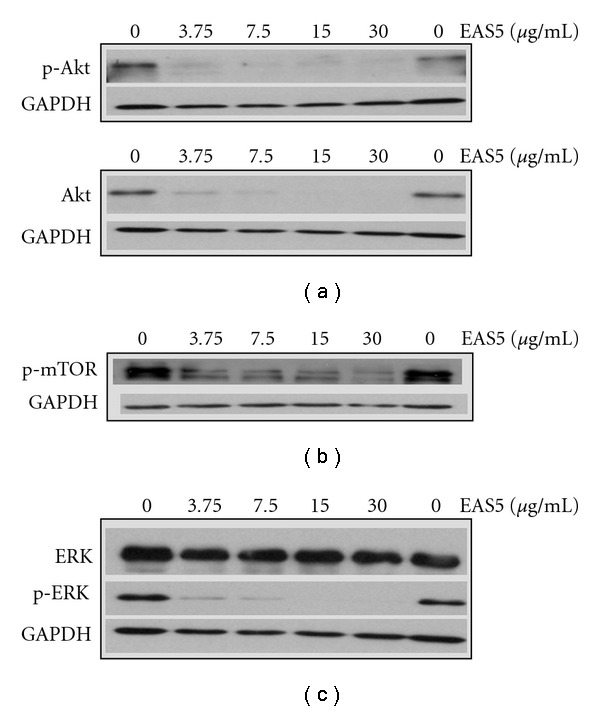
EAS5-induced inhibition of oncogenic signaling pathways in NB4 cells. Western blot analysis for phosphorylated (p-) and total Akt (a), phosphorylated mTOR (b), phosphorylated and total ERK (c) of EAS5-treated NB4 cells. Phosphorylated Akt, mTOR and ERK were all dramatically inhibited by treatment with EAS5. Total protein level of Akt was also dramatically decreased by EAS5, while the total ERK protein was not changed. The cells were treated with PBS or EAS5 (3.75–30 *μ*g/mL) for 72 h and then harvested. Cell lysates were then prepared for western blot analysis. EAS5: active fraction of THL.

Like THL, the EAS5-induced apoptosis of NB4 cells was also accompanied with the down-regulation of both PML-RAR*α* and DNMT1 proteins. At the dose of 3.75 *μ*g mL^−1^, EAS5 not only induced massive apoptosis but also diminished both PML-RAR*α* and DNMT1 of NB4 cells after 72 h treatment (Figure 12). These effects by EAS5 were schematically illustrated in Figure 7. At the same dose, EAS5 also markedly suppressed the Akt/mTOR and ERK signaling pathways in NB4 cells. EAS5 intensively decreased both the phosphorylated and total Akt protein levels (Figure 13(a)), and the phosphorylated mTOR was strongly suppressed by EAS5 as well (Figure 13(b)). Consistent with the effects of THL on the ERK of NB4 cells, EAS5 also dramatically decreased the ERK phosphorylation at the lowest dose of 3.75 *μ*g mL^−1^, however, the total ERK protein level was not affected even at the highest dose (Figure 13(c)). These effects of EAS5 on the oncogenic signaling pathways were illustrated in Figure 9.

## 4. Discussion

In the treatment of APL, the successful use of arsenic compounds contained in some traditional Chinese remedies revealed the potential of TCM in cancer therapy [3]. We used the APL cells as a model to evaluate the anticancer effects of another Chinese remedy THL, which is a herbal mixture consisting mainly of extracts from 14 Chinese medicinal herbs and has been used as an anticancer dietary supplement for more than 10 years. In this study, we comprehensively elucidated the molecular anticancer mechanisms of THL against APL cells in the aspects of apoptosis induction, cell-cycle regulation, down-regulation of PML-RAR*α* fusion protein and suppression of oncogenic signaling pathways.

Cell-cycle progression is closely related to the proliferation of cancer cells and therefore becomes the reasonable target for cancer therapy [5, 26]. There are several types of agents targeting the cell-cycle regulating proteins to arrest the cell cycle including chemotherapeutics. Chemotherapy agents such as taxol [27] and etoposide [28] induced G2/M arrest in cancer cells accompanied with the increase of cyclin B1. Similarly, ATO increased cyclin B1 in NB4 APL [7] and MCF-7 breast cancer [29] cells but decreased cyclin B1 in PCI-1 head and neck cancer cells during G2/M arrest induction [30]. In THL-induced G2/M arrest of NB4 leukemia cells, the cyclin B1 was also decreased rather than increased. This phenomenon was frequently observed in cancer cells treated with natural anticancer products, such as *Huanglian* [31], theaflavin (black tea polyphenol) [32], and genistein [33]. As THL is composed of a mixture of Chinese herbal extracts, it would be expected to decrease cyclin B1 during G2/M arrest induction.

THL decreased not only the cyclin B1 but also the cyclin A protein level of NB4 cells. This phenomenon was also seen in the G2/M arrest induced by other herbal extracts or phytochemicals, such as *Physalis angulata* extract [18] and carnosic acid, a polyphenol present in *Rosmarinus officinalis* [34]. In THL-treated NB4 cells, the cyclin A was suppressed by a smaller dose than cyclin B1. This phenomenon was similar to that induced by carnosic acid in Caco-2 colon cancer cells [34], which resulted in arresting the cell cycle prior to prometaphase. Therefore, it is speculated that the major cell-cycle arrest induced by THL may occur before prometaphase.

A hallmark of APL is a chromosomal translocation that results in the formation of PML-RAR*α* fusion protein, which blocks both the apoptosis and differentiation of APL cells [8]. According to our findings, THL induced dramatic apoptosis and down-regulation of PML-RAR*α* fusion protein in NB4 cells but no differentiation induction was observed (data not shown). Nason-Burchenal et al. [35] had demonstrated that cleavage of PML-RAR*α* mRNA by using a hammerhead ribozyme could trigger only apoptosis in NB4 APL cells but was insufficient to induce differentiation. Therefore, it is possible that THL may target the PML-RAR*α* mRNA by the similar manner, which remains to be explored.

Recent research has shown that the oncogenic effects of PML-RAR*α* fusion protein in leukemia were closely related to the hypermethylation on the promoters of target genes by recruiting DNMT [36]. DNMT1, the most important DNMT for maintaining the aberrant methylation [14], had been found to be aberrantly elevated not only in leukemia [9] but also in many types of cancer cells [37–42]. THL dramatically decreased both PML-RAR*α* fusion protein and DNMT1 during apoptosis induction. The decrease of DNMT1 may play an important role in THL-induced apoptosis of NB4 cells as it is reported that depletion of DNMT1 could induce or promote apoptosis of cancer cells [43, 44]. Inhibition of DNMT1 had been suggested as one of the targeted therapies for hematologic malignancies [45]. How to down-regulate DNMT1 appears as a sound approach for epigenetic cancer therapies [14, 46–48]. Considering its DNMT1 suppressing effect, THL might have potential as a candidate agent for epigenetic cancer therapy.

THL-induced apoptosis of NB4 cells may closely correlate with the inhibition of the oncogenic signaling pathways, such as Akt/mTOR, Stat3, and ERK. In acute leukemia cells, the Akt signaling is frequently activated and strongly contributes to proliferation and survival [21]. Constitutive Stat3 activation has been observed in a number of hematopoietic malignancies, and the apoptosis resistance in APL cells was hypothesized to be mediated through the augmentation of Stat3 signaling by the APL fusion proteins including the PML-RAR*α* [49]. The ERK signaling is frequently activated in AML cells and down-regulation of this aberrantly activated ERK had been shown to result in significant apoptosis of the AML blasts [20]. As targeting these three signaling pathways for effective leukemia therapy has been suggested [15], the inhibition of all these three cascades by THL further reveals its potentiality on APL therapy. Furthermore, the inhibitory effect of THL on ERK phosphorylation is notably dramatic. This may be useful for enhancing the therapeutic efficacy of another APL treating agent ATO. The ATO-induced apoptosis of acute leukemia cells is counteracted by the induction of ERK activity [50], and inhibition of ERK activity could enhance apoptosis in ATO-treated NB4 cells [51]. Therefore, it is speculated that combined or sequential use with THL plus ATO may provide the beneficial for APL treatment.

In addition to elucidate the comprehensive effects of THL against APL cells, we also purified the active fraction EAS5 from this herbal mixture. It seems that the active anticancer components in THL are relative hydrophobic, because the EAS5 was ethyl acetate soluble and eluted by ethyl acetate/*n*-hexane (80/20). This hydrophobic property of EAS5 may make it easier to be absorbed by GI tract. As the EAS5 was about one hundred times more potent than THL and exerted most of its multi-targeting effects against NB4 cells, it may be considered as a candidate for future clinical trial of complementary APL treatment.

## Funding

Formosa Cancer Foundation, Taipei, Taiwan and National Health Research Institutes, Zhunan, Taiwan (CA-097-PP-10).

## Figures and Tables

**Table 1:** The THL-induced G2/M arrest in NB4 cells.

Treatment	Cell-cycle distribution		
	Gl (%)	S (%)	G2/M (%)
PBS 24 h	48.36	32.43	19.21
PBS 48 h	48.01	28.72	23.27
THL1.5 mg/mL 24 h	11.45	38.27	50.28
THL 1.5 mg/mL 48 h	29.77	14.31	55.93
ATRA 0.25 *μ*M 24 h	53.90	29.27	16.83
ATRA 0.25 *μ*M 48 h	60.85	18.21	20.94

The cell-cycle distributions of THL- or ATRA-treated cells in Figure 3 were listed in the Table. THL induced G2/M arrest of cell cycle, in contrast to the G1 arrest induced by ATRA. THL, Tien-Hsien Liquid; ATRA, all-trans retinoic acid.

**Table 2:** Effects of EAS5 on the cell-cycle distribution of NB4 cells.

Treatment (*μ*g mL^−1^)	Cell-cycle distribution		
	Gl (%)	S (%)	G2/M (%)
PBS	56.93	28.62	14.44
EAS5 3.75	55.58	21.08	23.07
EAS5 7.5	48.76	29.60	21.64
EAS5 15	40.74	17.78	41.75
EAS5 30	41.63	14.22	44.14

The cell-cycle distributions of EAS5-treated NB4 cells in Figure 10(a) were listed in the Table. Like THL, EAS5 also induced G2/M arrest in NB4 cells. EAS5, active fraction of THL; THL, Tien-Hsien Liquid.

## References

[1] Sun A, Chia J-S, Wang W-B, Chiang C-P (2004). Immunomodulating effects of "Tien-Hsien liquid" on peripheral blood mononuclear cells and T-lymphocytes from patients with recurrent aphthous ulcerations. *American Journal of Chinese Medicine*.

[2] Sun A, Chia JS, Chiang CP, Hsuen SP, Du JL, Wu CW (2005). The Chinese herbal medicine Tien-Hsien liquid inhibits cell growth and induces apoptosis in a wide variety of human cancer cells. *Journal of Alternative and Complementary Medicine*.

[3] Chen G-Q, Zhu J, Shi X-G (1996). In vitro studies on cellular and molecular mechanisms of arsenic trioxide (As2O3) in the treatment of acute promyelocytic leukemia: As2O3 induces NB4 cell apoptosis with downregulation of Bcl-2 expression and modulation of PML- RAR*α*/PML proteins. *Blood*.

[4] Zhang T-D, Chen G-Q, Wang Z-G, Wang Z-Y, Chen S-J, Chen Z (2001). Arsenic trioxide, a therapeutic agent for APL. *Oncogene*.

[5] Senderowicz AM (2002). The cell cycle as a target for cancer therapy: basic and clinical findings with the small molecule inhibitors flavopiridol and UCN-01. *Oncologist*.

[6] Stewart ZA, Pietenpol JA (1999). Cell Cycle Checkpoints as Therapeutic Targets. *Journal of Mammary Gland Biology and Neoplasia*.

[7] Cai X, Yu Y, Huang Y (2003). Arsenic trioxide-induced mitotic arrest and apoptosis in acute promyelocytic leukemia cells. *Leukemia*.

[8] Villa R, Morey L, Raker VA (2006). The methyl-CpG binding protein MBD1 is required for PML-RAR*α* function. *Proceedings of the National Academy of Sciences of the United States of America*.

[9] Mizuno S-I, Chijiwa T, Okamura T (2001). Expression of DNA methyltransferases DNMT1, 3A, and 3B in normal hematopoiesis and in acute and chronic myelogenous leukemia. *Blood*.

[10] Chen GQ, Shi XG, Tang W, Xiong SM, Zhu J, Cai X (1997). Use of arsenic trioxide (As2O3) in the treatment of acute promyelocytic leukemia (APL): I. As2O3 exerts dose-dependent dual effects on APL cells. *Blood*.

[11] Zhu J, Lallemand-Breitenbach V, De Thé H (2001). Pathways of retinoic acid- or arsenic trioxide-induced PML/RAR*α* catabolism, role of oncogene degradation in disease remission. *Oncogene*.

[12] Cui X, Wakai T, Shirai Y, Yokoyama N, Hatakeyama K, Hirano S (2006). Arsenic trioxide inhibits DNA methyltransferase and restores methylation-silenced genes in human liver cancer cells. *Human Pathology*.

[13] Fazi F, Travaglini L, Carotti D (2005). Retinoic acid targets DNA-methyltransferases and histone deacetylases during APL blast differentiation in vitro and in vivo. *Oncogene*.

[14] Robert M-F, Morin S, Beaulieu N (2003). DNMT1 is required to maintain CpG methylation and aberrant gene silencing in human cancer cells. *Nature Genetics*.

[15] McCubrey JA, Steelman LS, Abrams SL (2008). Targeting survival cascades induced by activation of Ras/Raf/MEK/ERK, PI3K/PTEN/Akt/mTOR and Jak/STAT pathways for effective leukemia therapy. *Leukemia*.

[16] Steelman LS, Abrams SL, Whelan J (2008). Contributions of the Raf/MEK/ERK, PI3K/PTEN/Akt/mTOR and Jak/STAT pathways to leukemia. *Leukemia*.

[17] Clemens DL, Calisto LE, Sorrell MF, Tuma DJ (2003). Ethanol metabolism results in a G2/M cell-cycle arrest in recombinant Hep G2 cells. *Hepatology*.

[18] Hsieh W-T, Huang K-Y, Lin H-Y, Chung J-G (2006). Physalis angulata induced G2/M phase arrest in human breast cancer cells. *Food and Chemical Toxicology*.

[19] Kawasaki A, Matsumura I, Kataoka Y, Takigawa E, Nakajima K, Kanakura Y (2003). Opposing effects of PML and PML/RAR*α* on STAT3 activity. *Blood*.

[20] Lunghi P, Tabilio A, Dall’Aglio PP (2003). Downmodulation of ERK activity inhibits the proliferation and induces the apoptosis of primary acute myelogenous leukemia blasts. *Leukemia*.

[21] Martelli AM, Nyåkern M, Tabellini G (2006). Phosphoinositide 3-kinase/ Akt signaling pathway and its therapeutical implications for human acute myeloid leukemia. *Leukemia*.

[22] Récher C, Dos Santos C, Demur C, Payrastre B (2005). mTOR, a new therapeutic target in acute myeloid leukemia. *Cell Cycle*.

[23] Chow S, Minden MD, Hedley DW (2006). Constitutive phosphorylation of the S6 ribosomal protein via mTOR and ERK signaling in the peripheral blasts of acute leukemia patients. *Experimental Hematology*.

[24] Dos Santos C, Récher C, Demur C, Payrastre B (2006). The PI3K/Akt/mTOR pathway: a new therapeutic target in the treatment of acute myeloid leukemia. *Bulletin du Cancer*.

[25] Smits VAJ, Klompmaker R, Vallenius T, Rijksen G, Mäkelä TP, Medema RH (2000). p21 Inhibits Thr161 phosphorylation of Cdc2 to enforce the G2 DNA damage checkpoint. *Journal of Biological Chemistry*.

[26] Carnero A (2002). Targeting the cell cycle for cancer therapy. *British Journal of Cancer*.

[27] Ling Y-H, Consoli U, Tornos C, Andreeff M, Perez-Soler R (1998). Accumulation of cyclin B1, activation of cyclin B1-dependent kinase and induction of programmed cell death in human epidermoid carcinoma KB cells treated with taxol. *International Journal of Cancer*.

[28] Sleiman RJ, Stewart BW (2000). Early caspase activation in leukemic cells subject to etoposide-induced G2-M arrest: evidence of commitment to apoptosis rather than mitotic cell death. *Clinical Cancer Research*.

[29] Ling Y-H, Jiang J-D, Holland JF, Perez-Soler R (2002). Arsenic trioxide produces polymerization of microtubules and mitotic arrest before apoptosis in human tumor cell lines. *Molecular Pharmacology*.

[30] Seol JG, Park WH, Kim ES (1999). Effect of arsenic trioxide on cell cycle arrest in head and neck cancer cell line PCI-1. *Biochemical and Biophysical Research Communications*.

[31] Li XK, Motwani M, Tong W, Bornmann W, Schwartz GK (2000). Huanglian, A Chinese herbal extract, inhibits cell growth by suppressing the expression of cyclin B1 and inhibiting CDC2 kinase activity in human cancer cells. *Molecular Pharmacology*.

[32] Prasad S, Kaur J, Roy P, Kalra N, Shukla Y (2007). Theaflavins induce G2/M arrest by modulating expression of p21waf1/cip1, cdc25C and cyclin B in human prostate carcinoma PC-3 cells. *Life Sciences*.

[33] Choi YH, Zhang L, Lee WH, Park K-Y (1998). Genistein-induced G2/M arrest is associated with the inhibition of cyclin B1 and the induction of p21 in human breast carcinoma cells. *International Journal of Oncology*.

[34] Visanji JM, Thompson DG, Padfield PJ (2006). Induction of G2/M phase cell cycle arrest by carnosol and carnosic acid is associated with alteration of cyclin A and cyclin B1 levels. *Cancer Letters*.

[35] Nason-Burchenal K, Takle G, Pace U, Flynn S, Allopenna J, Martin P (1998). Targeting the PML/RAR alpha translocation product triggers apoptosis in promyelocytic leukemia cells. *Oncogene*.

[36] Di Croce L, Raker VA, Corsaro M (2002). Methyltransferase recruitment and DNA hypermethylation of target promoters by an oncogenic transcription factor. *Science*.

[37] Agoston AT, Argani P, Yegnasubramanian S (2005). Increased protein stability causes DNA methyltransferase 1 dysregulation in breast cancer. *Journal of Biological Chemistry*.

[38] Etoh T, Kanai Y, Ushijima S (2004). Increased DNA methyltransferase 1 (DNMT1) protein expression correlates significantly with poorer tumor differentiation and frequent DNA hypermethylation of multiple CpG islands in gastric cancers. *American Journal of Pathology*.

[39] McCabe MT, Davis JN, Day ML (2005). Regulation of DNA methyltransferase 1 by the pRb/E2F1 pathway. *Cancer Research*.

[40] Nakagawa T, Kanai Y, Ushijima S, Kitamura T, Kakizoe T, Hirohashi S (2005). DNA hypermethylation on multiple CpG islands associated with increased DNA methyltransferase DNMT1 protein expression during multistage urothelial carcinogenesis. *The Journal of Urology*.

[41] Peng D-F, Kanai Y, Sawada M (2005). Increased DNA methyltransferase 1 (DNMT1) protein expression in precancerous conditions and ductal carcinomas of the pancreas. *Cancer Science*.

[42] Sawada M, Kanai Y, Arai E, Ushijima S, Ojima H, Hirohashi S (2007). Increased expression of DNA methyltransferase 1 (DNMT1) protein in uterine cervix squamous cell carcinoma and its precursor lesion. *Cancer Letters*.

[43] Kassis ES, Zhao M, Hong JA, Chen GA, Nguyen DM, Schrump DS (2006). Depletion of DNA methyltransferase 1 and/or DNA methyltransferase 3b mediates growth arrest and apoptosis in lung and esophageal cancer and malignant pleural mesothelioma cells. *Journal of Thoracic and Cardiovascular Surgery*.

[44] Zhang S, Zeng F, Peng S, Zhu C, Li H, Wang L (2009). Effects on biological behavior of bladder carcinoma T24 cells via silencing DNMT1 and/or DNMT3b with shRNA in vitro. *Journal of Huazhong University of Science and Technology*.

[45] Bhalla KN (2005). Epigenetic and chromatin modifiers as targeted therapy of hematologic malignancies. *Journal of Clinical Oncology*.

[46] Hwan SC, Jong HP, Kim Y-J (2007). Epigenomics: novel aspect of genomic regulation. *Journal of Biochemistry and Molecular Biology*.

[47] Jung Y, Park J, Kim TY (2007). Potential advantages of DNA methyltransferase 1 (DNMT1)-targeted inhibition for cancer therapy. *Journal of Molecular Medicine*.

[48] Reid GK, Besterman JM, MacLeod AR (2002). Selective inhibition of DNA methyltransferase enzymes as a novel strategy for cancer treatment. *Current Opinion in Molecular Therapeutics*.

[49] Dong S, Chen S-J, Tweardy DJ (2003). Cross-talk between retinoic acid and stat3 signaling pathways in acute promyelocytic leukemia. *Leukemia and Lymphoma*.

[50] Bonati A, Rizzoli V, Lunghi P (2006). Arsenic trioxide in hematological malignancies: the new discovery of an ancient drug. *Current Pharmaceutical Biotechnology*.

[51] Lunghi P, Tabilio A, Lo-Coco F, Pelicci PG, Bonati A (2005). Arsenic trioxide (ATO) and MEK1 inhibition synergize to induce apoptosis in acute promyelocytic leukemia cells. *Leukemia*.

